# The Strand Palace Hotel

**Published:** 1909-09-11

**Authors:** 


### THE STRAND PALACE HOTEL.

This, the newest hotel in London, presents many features of interest both from an administrative and from a constructive point of view. Standing on a fine central site, on the spot where formerly stood Exeter Hall, it has a comparatively small frontage, which hardly gives a fair impression of the size and palatial outlines of the building itself. There are 470 bedrooms, all comfortably furnished and with hot and cold water laid on. Special attention has been paid to the ventilation of the hotel. The whole building is warmed by means of radiators in winter, ventilation being ensured by electric-driven fans and extractors, and the fresh air supplied being previously washed by being drawn through running water. This, we believe, is the first time the system has been applied to a London hotel, and its results are to be appreciated by visitors who make use of the Strand Palace during the foggy winter days. Adequate fire provision is ensured by fireproof partitions screening the various corridors, and the whole building is practically fire-resisting. A water reservoir with a capacity of 60,000 gallons is provided as a reserve on the roof. From a hygienic point of view the construction of the new hotel leaves nothing to be desired. The building will be opened to the public early next week, and the management offers many attractions to the public. Not the least of these is the "no tip" system which is to prevail —an innovation which will have the cordial support of the majority of visitors.

